# Genetic Characterization of the “Chusca Lojana”, a Creole Goat Reared in Ecuador, and Its Relationship with Other Goat Breeds

**DOI:** 10.3390/ani10061026

**Published:** 2020-06-12

**Authors:** Lenin Aguirre-Riofrio, Teddy Maza-Tandazo, Manuel Quezada-Padilla, Oscar Albito-Balcazar, Alex Flores-Gonzalez, Osvaldo Camacho-Enriquez, Amparo Martinez-Martinez, BioGoat Consortium, Juan Vicente Delgado-Bermejo

**Affiliations:** 1 Agricultural Faculty, Veterinary Medicine and Zootechnics School, National University of Loja, Loja 110110, Ecuador; temata1@hotmail.com (T.M.-T.); manuel.quezada@unl.edu.ec (M.Q.-P.); oscar.albito@unl.edu.ec (O.A.-B.); 2 Graduation Students in Veterinary Medicine and Zootechnics, National University of Loja, Zapotillo 110901, Ecuador; afloresg@mag.gob.ec (A.F.-G); vladimirenrriquez@hotmail.com (O.C.-E.); 3 Department of Genetics, University of Cordoba, 14014 Cordoba, Spain; amparomartinezuco@gmail.com (A.M.-M.); id1debej@uco.es (J.V.D.-B.); 4 Latin American Goat Biodiversity Project, Department of Genetics, University of Cordoba, 14014 Cordoba, Spain; ib2mamaa@uco.es

**Keywords:** *Capra hircus*, biodiversity, genetic resources, conservation, microsatellites markers

## Abstract

**Simple Summary:**

An individual from a population presents a series of characteristics that differ from the rest and that increase as the kinship relationships are lower; this leads to the fact that two populations that stop exchanging genetic material through mating eventually come to present characters common to all members of each, but different between the two. This was what happened with the animal populations brought to America more than 500 years ago from the Iberian Peninsula in the colonization period, resulting in Creole populations that inhabit the harshest environments of our immense geography. The Creole goat “Chusca Lojana” has adapted to live in the dry forest region of Southern Ecuador, where environmental conditions are warm-dry, with sparse vegetation and a rather irregular topography. In the present study, the intra-breed genetic diversity of this goat is analyzed as well as its genetic relationships with other breeds. Significant F_IS_ and intra-breed structure show that there is some heterogeneity and structure within the breed. However, inter-breed structure results underline that this breed is differentiated from other Creole breeds, because crossbreeding with other breeds was not detected; therefore, we must take advantage of this valuable genetic resource, and ensure its conservation and selection.

**Abstract:**

The largest population of goats (62%) in Ecuador is in the dry forest region in the south of the country. A Creole goat, named “Chusca Lojana”, has adapted to the dry forest region where environmental conditions are warm-dry, with sparse vegetation. Knowledge of the genetic information of the Creole goat is important to determine intra-racial diversity, the degree of genetic distance among other breeds of goats, and the possible substructure of the population, which is valuable for the conservation of such a species’ genetic resources. A total of 145 samples of the Creole goat was taken from the four biotypes previously identified. Genetic analyses were performed using 38 microsatellites recommended for studies of goat genetic diversity (FAO-ISAG). The results of within-breed genetic diversity showed a mean number of alleles per locus (MNA) of 8, an effective number of alleles (Ae) of 4.3, an expected heterozygosity (He) of 0.71, an observed heterozygosity (Ho) of 0.63, polymorphic information content (PIC) of 0.67, and an F_IS_ value of 0.11. Between-breed genetic diversity among 43 goat populations (native of Spain, American Creole, Europeans, and Africans) showed the following values: F_IS_ = 0.087, F_IT_ = 0.176, and F_ST_ = 0.098. Regarding the analysis of the population structure, the results showed that the Creole Chusca Lojana goat population is homogeneous and no genetic separation was observed between the different biotypes (F_ST_ = 0.0073). In conclusion, the Chusca Lojana goat has a high genetic diversity, without exhibiting a genetic substructure. Therefore, it should be considered as a distinct population because crossbreeding with other breeds was not detected.

## 1. Introduction

Biodiversity and abiotic factors are responsible for maintaining the balance and stability of ecosystems. According to [1], biodiversity is the sum of all living organisms on earth, comprising the wide variety of species, ecosystems, and ecological processes that make up our planet. Therefore, biological diversity in a particular place, region, or country must be considered as a key resource, not only for its genetic heritage, but also for its ecological, social, economic, scientific, educational, cultural, and aesthetic values.

In the last two decades, however, such genetic heritage has been jeopardized and more than one million species have become endangered [2,3]. Regarding livestock species, some of the local breeds (26%) of animals in the world are currently endangered, 7% of which has already disappeared only in the last 20 years [4]. Replacement of local breeds by exotic ones may involve an increase in the degree of inbreeding in these populations [5,6], with a subsequent decrease in effective population size [7] and a decline in resistance and resilience to environmental changes.

Goat farming is of great significance for the economy of rural populations worldwide, especially in developing countries due to the rusticity of goats [6,8]. Goats are capable of taking advantage of low-quality natural resources that are unsuitable to other species [9]. This is the case of the Creole goat “Chusca Lojana”, which has adapted to the Dry Forest region in the Loja Province of Ecuador, an environment with a pronounced dry season during parts of the year and limited natural resources. Against this background, the purpose of the present study was to characterize the Creole Chusca Lojana goat genetically for future genetic conservation, use, and management, being a priority task because this resource represents a genetic, cultural, social, and economic heritage for this region.

## 2. Materials and Methods

### 2.1. Samples and DNA Extraction

Hair samples were obtained from 145 Chusca Lojana goats of four different biotypes, the same ones that differ from each other in the size and shape of the ears (“oreja corta y doblada”, “oreja de leon”, and “oreja torneada” biotypes) and in the absence of horns (“muco” biotype). Sampled individuals belonged to different herds and locations and kinship was minimized, so genetic variability of sampling was ensured. Samples were collected in paper envelopes with individual information and stored at room temperature until genetic analyses were performed at the Laboratory of Applied Molecular Genetics of the University of Cordoba, Spain. Sampling was carried out by qualified veterinarians during routine technical assistance, so the approval of an animal ethics and welfare committee for biological sampling collection was not required. The genomic DNA of the hair follicle samples was extracted according to the methodology described by [10].

### 2.2. Genotyping Using STR Markers

Thirty-eight microsatellites were used, including the thirty recommended by the FAO/ISAG (Food and Agriculture Organization of the United Nations/International Society of Animal Genetics) for genetic diversity studies in goat species. The microsatellites included were *BM1258*, *BM1329, BM1818, BM6506, BM6526, BM8125, CSRD247, CSRM60, CSSM66, ETH010, ETH225, HAUT27, HSC, ILSTS008, ILSTS011, ILSTS019, ILSTS030, ILSTS087, INRA005, INRA006, INRA023, INRA063, INRA172, MAF065, MAF209, McM527, MM12, OarFCB011, OarFCB020, OarFCB048, OarFCB304, SPS115, SRCRSP05, SRCRSP08, SRCRSP23, SRCRSP24, TGLA053,* and *TGLA122*. After amplification by the polymerase chain reaction (PCR) technique, size separation of the amplified fragments was carried out by polyacrylamide gel electrophoresis in an ABI 3130XL automatic sequencer by means of Genescan^®^ 400HD ROX Size Standard (Fisher Scientific, Madrid, Spain). Fragment analysis and allelic typing were performed using Genescan Analysis^®^ 3.1.2 (Fisher Scientific, Madrid, Spain) and Genotyper^®^ 2.5.2 software (Fisher Scientific, Madrid, Spain), respectively (Figure S1).

### 2.3. Genetic Diversity Within-Breed

The MICROSATELLITE TOOLKIT software for Excel [11] was used to determine the average number of alleles per locus (MNA), expected allelic frequencies (He), observed heterozygosis (Ho), and polymorphic information content (PIC). The effective number of alleles was calculated with the PopGene program [12]. The F_IS_ fixation index values with a 95% confidence interval were calculated with the GENETIX v software 4.05 [13]. The Hardy–Weinberg (HW) equilibrium test was performed using the GENEPOP v program 3.1c [14], which applies Fisher’s exact test using the Monte Carlo Markov chain method [15] and Bonferroni correction.

### 2.4. Genetic Diversity Inter-Breed

To obtain the genetic differentiation, structure, and distance information data of the Chusca Lojana goat was compared with data of other 42 goat populations worldwide, which included some native Creole breeds from Spain, Europe, America, and Africa (Table 1). Data on worldwide breeds were collected from the online database of the Laboratory of Applied Molecular Genetics and the BioGoat Consortium (https://biogoat.jimdo.com/razas-breeds/).

Wright’s F statistics, namely F_IT_, F_ST_, and F_IS_ [16], were calculated using GENETIX software [13]. A Factorial Correspondence Analysis was performed with the same program. The Reynolds’ genetic distance [17] was determined using the POPULATIONS software [18]. With the distance values obtained, a Neighbor-Net was carried out using the SPLITSTREE program [19] to graphically represent the genetic relationships among the breeds. Table 1 shows the compared goat populations, their origin, and the number of samples analyzed for each population, in which 24 microsatellites common to all breeds were identified.

### 2.5. Genetic Structure of Chusca Lojana Goat

The genetic distances between individuals (D_SA_) were calculated [20], and a dendrogram was elaborated using the TREEVIEW program [21]. An analysis of the substructure of the Creole Chusca Lojana goat was also carried based on a Bayesian algorithm with the STRUCTURE v 2.1 program [22], which uses a model based on the Monte Carlo Markov chain method to estimate the consequent distribution of the admixture proportion of each individual (q). The analysis was performed using a 100,000 burn-in period followed by 300,000 iterations of Monte Carlo repeating each run 10 times.

## 3. Results

### 3.1. Genetic Diversity Within-Breed

Shown in Table 2 are the values obtained for expected heterozygosity (He), observed heterozygosity (Ho), polymorphic information content (PIC), the F_IS_ values with their standard deviations, and the deviated markers of the Hardy–Weinberg equilibrium. Given the PIC values obtained, most markers were informative (PIC > 0.5). After Bonferroni correction, only the INRA023 and SRCRSP24 markers were unstable compared with the Hardy–Weinberg equilibrium of the population. Only the INRA005 and SPS115 markers showed a homozygous defect, but their F_IS_ values were not significant. Half of the microsatellites (*n* = 19) showed a significant excess of homozygosity, and the other microsatellites (*n* = 19) displayed F_IS_ values that were not significantly different from 0.

The mean number of alleles (MNA) is an indicator of the genetic variability within populations. The MNA is 8.26 in the Chusca Lojana goat, and with an average Ae value of 4.25. We assessed the genetic diversity within a population by calculating the proportion of heterozygous individuals or heterozygosity. In Table 2, the values of expected mean heterozygosity (He = 0.706) and mean heterozygosity by direct count (Ho = 0.629) are shown. The value of F_IS_ with a 95% confidence interval and 1000 randomizations was significant (F_IS_ = 0.1099 (0.08773–0.12549)), which indicates that the population could deviate from the Hardy–Weinberg equilibrium.

### 3.2. Genetic Structure of the Chusca Lojana Goat

**Figure 1:**
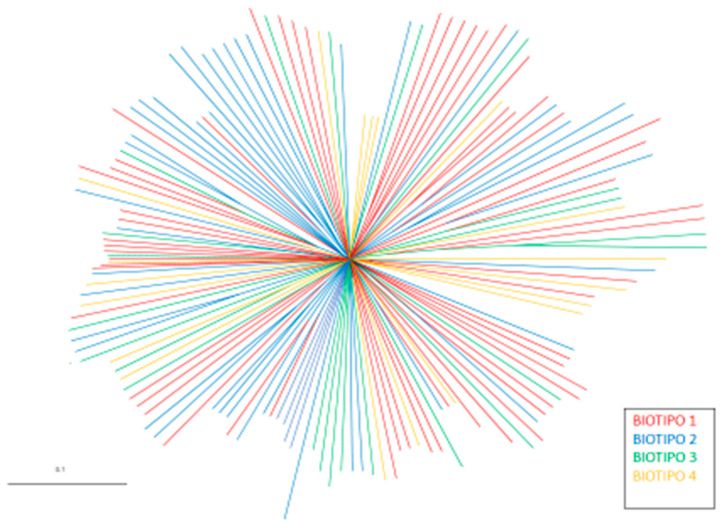
Tree diagram of the individual genetic distances (D_SA_).

To make a preliminary assessment of the population’s homogeneity, a tree diagram of the genetic distances between individuals was designed (Figure 1), in which the individuals are grouped with color codes according to their genetic proximity. There is no clear grouping of the individuals by biotypes. This lack of genetic separation is supported by a very low genetic differentiation among them (F_ST_ = 0.0073).

**Figure 2:**
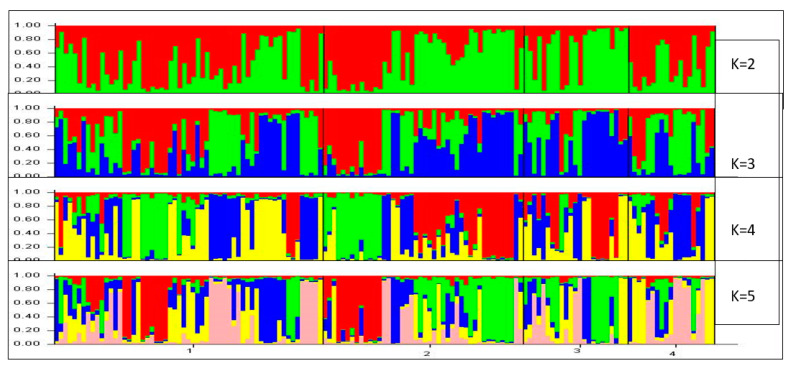
Genetic structure of the Chusca Lojana. Cluster diagram of the population for K ranging from 2 to 5.

A more complex analysis of the population structure was also carried out using STRUCTURE v. 2.1. This software [22] allowed the calculation of the admixture proportions of each individual (q). The mean distribution represents an estimation of the proportion of the genome that every individual displayed in relation to the parental population. A grouping analysis of the individuals was also carried out with a different number of clusters (K) representing the assumed number of populations. An admixture model was designed using the above software, in which each individual could contain in its genome a different percentage of the ancestral populations from which it might come. When only the Chusca Lojana goat was considered, heterogeneous genetic structure was detected in the four biotypes (Figure 2).

### 3.3. Genetic Diversity Inter-Breed

The genetic differentiation among the 43 populations of goats included in the present study was high, with the following statistical values for F: F_IS_ = 0.0871 (0.065–0.116), F_IT_ = 0.176 (0.154–0.205), and F_ST_ = 0.0978 (0.089–0.108).

**Figure 3:**
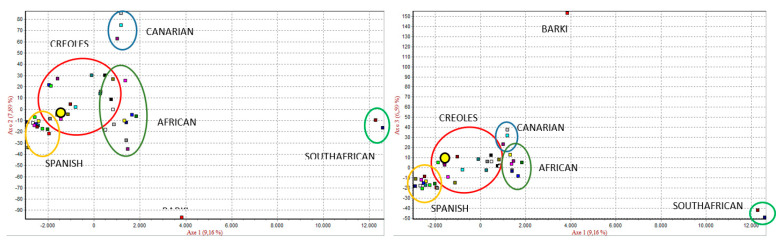
Correspondence Factor Analysis of 43 goat populations.

The results of the Correspondence Factor Analysis (Figure 3) show that the South African breeds (Axis 1) and the Egyptian Barki breed (Axis 2) are different from the others. The Chusca Lojana goat, which is represented by a yellow circle, is related to other Creole breeds.

The Reynolds’ genetic distance data and the F_ST_ values between pairs of goat populations are shown in Table S1. The values for the Chusca Lojana goat are highlighted in gray, showing the lowest values of genetic distance with the other Creole goats (0.03–0.11) and the largest distance with the Galapagos goat (0.18) and the South African goats (Kalahari, 0.15; and Boer, 0.18).

**Figure 4:**
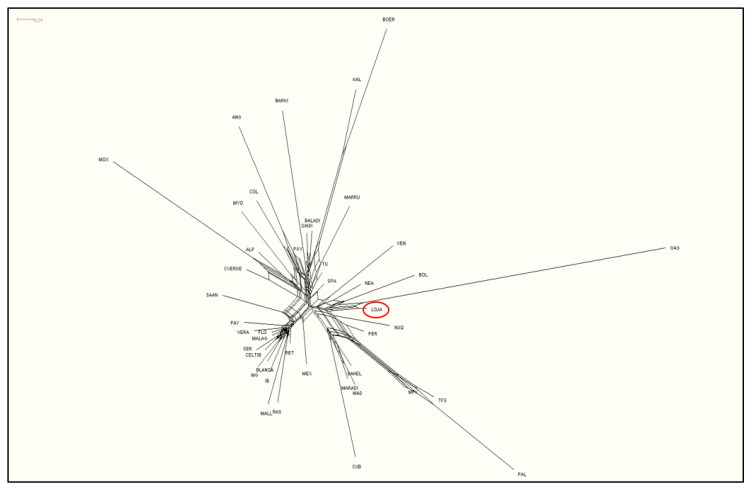
Neighbor-Net representation of Reynolds’ genetic distances between 43 goat populations.

In the graphic representation of Reynolds’ genetic distances in a network dendrogram (Figure 4), it can be observed that the Chusca Lojana and the Galapagos Islands breeds share similar origins with the Bolivian Creole Goats.

**Figure 5:**
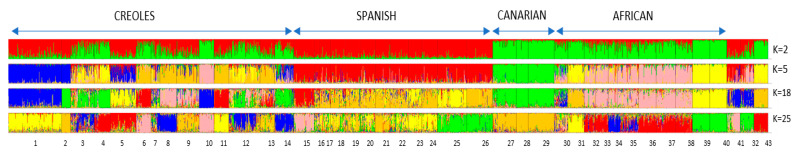
Genetic structure of the 43 goat populations analyzed. Graphical representation of clusters when K = 2, K = 5, K = 18, and K = 25. 1: Chusca Lojana; 2: Galapagos goat; 3: Spanish goat; 4: Myotonic; 5: Mexicana; 6: Creole from Cuba; 7: Creole from Colombia; 8: Creole from Venezuela; 9: Creole from Perú; 10: Moxotó; 11: Creole from Bolivia; 12: Creole from Paraguay; 13: North-east creole; 14: Neuquina; 15: Blanca de Rasquera; 16: Retinta; 17: Verata; 18: Blanca Andaluza; 19: Celtibérica; 20: Malagueña; 21: Murciano-Granadina; 22: Florida; 23: Payoya; 24: Negra Serrana; 25: Pitiusa; 26: Mallorquina; 27: Majorera; 28: Palmera; 29: Tinerfeña; 30: Cabo Verde; 31: Barki; 32: Baladi; 33: Saidi; 34: Morocco; 35: Tunisian Local Goat; 36: Maradi; 37: West African Dwarf; 38: Sahel; 39: Kalahari Goat; 40: Boer; 41: Saanen; 42: Alpina; 43: Anglo-Nubiana.

Shown graphically in Figure 5 is the population structure of the 43 goat populations using STRUCTURE v.2.1 software. Every individual is represented by a vertical bar and each color is a uniform proportion of the corresponding cluster. When the number of estimated populations is 2 (K = 2), data are separated in two clusters, in which one group corresponds to the European breeds (Spanish, Saanen, and Alpine) and most of the Creole breeds (shown with red), and the other group includes the African and Canary Islands breeds (shown with green). When K = 5, the breeds are separated into several clusters, such as the Spanish, the Canary Islands, and the African breeds. When K = 18, the Chusca Lojana goat is separated from the other Creole goat populations. Statistically, the optimal number of populations is K = 25. There is no subdivision or substructure of the Chusca Lojana goat when K > 18. From K = 2, more than 80% of the analyzed individuals are assigned to a single cluster.

## 4. Discussion

The characterization process is the first step of the zoogenetic resources conservation programs. So, studies of this nature are generally recommended to save locally adapted breeds from extinction [23].

The high genetic diversity of the Chusca Lojana breed is demonstrated by the mean number of alleles (MNA = 8.3) and the effective number of alleles (Ae = 4.3). These values are similar to those obtained for the Spanish goat of the USA (7.81 and 4.24, respectively) and higher than those of the Creole goats of Argentina, Bolivia, Brazil, Colombia, Cuba, Peru, and Venezuela (MNA = 5.24–6.81; Ae = 2.61–3.87). It is noteworthy that the Galapagos Creole goat displayed lower MNA and Ae values (MNA = 3.05, Ae = 1.98), and the Creole Goat of Paraguay showed higher MNA and Ae values (8.71 and 5.19, respectively) [24].

The Chusca Lojana breed exhibited higher expected (He = 0.71) and observed heterozygosity values (Ho = 0.63) than the mean of the corresponding values for the other American Creole goat populations (He = 0.638, Ho = 0.585). The only exception was the Paraguayan Creole, which showed higher He and Ho values [24]. The genetic diversity of the Chusca Lojana breed is similar to that reported for animals from the Central and Eastern Mediterranean (He = 0.737 and Ho = 0.663) [25], but slightly higher than the diversity manifested in native breeds of the Iberian Peninsula (He = 0.65 and Ho = 0.61) [26] and in 71 populations in Africa and America (MNA = 6, He = 0.64 and Ho = 0.6) [27]. The F_IS_ value of the Chusca Lojana breed was significant (0.1098) and higher than the mean of the corresponding values reported for the other American goat populations [24,27], being the values 0.083 and 0.082, respectively. This significant F_IS_ value could be due to non-random mating within the breed. Significant inbreeding levels and deviations from the Hardy–Weinberg equilibrium can be shown in a short time if no measures are taken.

Interestingly, the Chusca Lojana breed showed higher genetic diversity (MNA = 8.3, He = 0.71, and Ho = 0.63) than reported for 57 native goat breeds in Asia (MNA = 5.98, He = 0.59, and Ho = 0.54) [28]. However, the F_IS_ of the 57 Asian breeds was lower (0.073) and the F_IT_ = 0.191 was similar to the corresponding values in the present study.

The genetic differentiation (F_ST_) of the Chusca Lojana breed and the other 42 Creole and transboundary breed populations was lower (F_ST_ = 0.098) than the values reported for 24 Creole and three cross-border breeds in America ([24], F_ST_ = 0.134; [27], F_ST_ = 0.13), for the Creole breeds of Asia (F_ST_ = 0.127) [28], and for the Creole goats of Cuba, comprising the Iberic and the African breeds (F_ST_ = 0.112) [29]. On the other hand, the F_ST_ of the Chusca Lojana breed was higher than the values obtained for goats from Northern Europe and Central-Eastern Mediterranean (0.07) [25], West Asia (0.075) [30], and Africa (0.071) [31].

By graphic representation of Reynolds’ genetic distances, it could be demonstrated that results in the present study are in accordance with the data previously reported [27], in which the Creole goat of Ecuador was grouped in the same cluster with the Bolivian, Venezuelan, Northwest Argentinian, and Peruvian goats. However, that is not the case in the results described by [24], in which the Ecuadorian goat was found to be genetically related to the Anglo Nubian, Colombian, and Paraguayan breeds, but unrelated to the Galapagos and the Bolivian goats. Therefore, it is likely that the Creole goat population studied by [24] do not correspond to the goat population Chusca Lojana.

Finally, both the work carried out by [27] and the present study evaluated similar populations and represented their population structure graphically using the STRUCTURE program. As a result of the analyses of genetic dispersion by mean of the geographic cluster model, the origins of several groups of goat breeds, such as the Iberic, Canarian, African, and American Creole, were confirmed. Accordingly, the latter is ancestrally related to the Iberian and African goat breeds.

## 5. Conclusions

The Chusca Lojana goat breed exhibits a high genetic diversity, with a heterogeneous structure inside the population; but, the existence of four different biotypes empirically admitted inside the breed was not supported by our genetic results. The Chusca Lojana breed belongs to the genetic group of the American Creoles, descendants of the of the Iberian populations imported by the Spanish colonizers five centuries ago. No genetic introgressions from the most important international breeds (i.e., Saanen, Alpina, Anglo-Nubian, African, and/or Spanish breeds) were detected in the Chusca Lojana breed.

The Chusca Lojana breed is a valuable zoogenetic resource, well adapted to its difficult environment; its conservation and selection should be encouraged, and actions should be taken to avoid future genetic erosion.

## Figures and Tables

**Table 1:** Populations studied, acronym, origin, and number of samples analyzed for each population.

	Breed/Population	Acronym	Origin	n
1	Chusca Lojana	LOJ	Ecuador	145
2	Galapagos goat	GAG	Galapagos Islands (Ecuador)	24
3	Spanish goat	SPA	USA	64
4	Myotonic	MYO	USA	43
5	Mexicana	MEX	Mexico	70
6	Creole from Cuba	CUB	Cuba	40
7	Creole from Colombia	COL	Colombia	24
8	Creole from Venezuela	VEN	Venezuela	45
9	Creole from Perú	PER	Peru	61
10	Moxotó	MOX	Brazil	40
11	Creole from Bolivia	BOL	Bolivia	40
12	Creole from Paraguay	PGY	Paraguay	84
13	North-east creole	NEA	Argentina	40
14	Neuquina	NUQ	Argentina	51
15	Blanca de Rasquera	RAS	Spain	56
16	Retinta	RET	Spain	15
17	Verata	VERA	Spain	30
18	Blanca Andaluza	BLANCA	Spain	40
19	Celtibérica	CELTIB	Spain	40
20	Malagueña	MALAG	Spain	40
21	Murciano-Granadina	MG	Spain	40
22	Florida	FLO	Spain	50
23	Payoya	PAY	Spain	36
24	Negra Serrana	SER	Spain	42
25	Pitiusa	IB	Islas Baleares (Spain)	79
26	Mallorquina	MALL	Islas Baleares (Spain)	70
27	Majorera	MFV	Islas Canarias (Spain)	64
28	Palmera	PAL	Islas Canarias (Spain)	32
29	Tinerfeña	TF2	Islas Canarias (Spain)	70
30	Cabo Verde	CVERDE	Cabo Verde	37
31	Barki	BARKI	Egypt	44
32	Baladi	BALADI	Egypt	31
33	Saidi	SAIDI	Egypt	34
34	Morocco	MOR	Marruecos	24
35	Tunisian Local Goat	TU	Tunisia	58
36	Maradi	MARADI	Nigeria	47
37	West African Dwarf	WAD	Nigeria	52
38	Sahel	SAHEL	Nigeria	46
39	Kalahari Goat	KAL	South Africa	47
40	Boer	BOER	South Africa	46
41	Saanen	SAAN	International	36
42	Alpina	ALP	International	37
43	Anglo-Nubiana	ANG	International	41

**Table 2:** Genetic results of the “Chusca Lojana” goat: microsatellites, mean number of alleles (MNA), effective number of alleles (Ae), expected heterozygosity (He), observed heterozygosity (Ho), polymorphic information content (PIC), F_IS_ values and confidence interval, and Hardy–Weinberg equilibrium (HWE) deviations.

Microsatellites	MNA	Ae	He	Ho	PIC	FIS	FIS IC	HWEd
*BM1258*	12	5.04	0.804	0.755	0.78	0.06116	(−0.02427–0.13910)	NS
*BM1329*	8	5.99	0.836	0.741	0.81	0.11373	(−0.02560–0.19802)	NS
*BM1818*	9	4.65	0.788	0.746	0.76	0.05246	(−0.02859–0.13614)	NS
*BM6506*	11	6.73	0.855	0.746	0.84	0.12696	(0.04177–0.20820)	NS
*BM6526*	9	5.65	0.826	0.819	0.8	0.008	(−0.06846–0.07866)	NS
*BM8125*	7	2.35	0.576	0.559	0.53	0.03099	(−0.08580–0.14336)	NS
*CSRD247*	7	4.61	0.786	0.729	0.76	0.07246	(−0.01127–0.15292)	NS
*CSRM60*	8	4.43	0.777	0.748	0.74	0.03741	(−0.05035–0.12679)	NS
*CSSM66*	20	7.02	0.861	0.69	0.84	0.1987	(0.11256–0.27766)	NS
*ETH010*	4	2.06	0.516	0.434	0.44	0.15805	(0.00882–0.30514)	NS
*ETH225*	6	1.30	0.233	0.214	0.22	0.08139	(−0.06547–0.23836)	NS
*HAUT27*	7	3.68	0.731	0.622	0.7	0.14921	(0.05547–0.23469)	NS
*HSC*	14	8.42	0.885	0.789	0.87	0.10785	(0.03307–0.18731)	NS
*ILSTS008*	3	1.60	0.375	0.352	0.34	0.06315	(−0.06421–0.19046)	NS
*ILSTS011*	8	1.95	0.49	0.469	0.43	0.04338	(−0.08673–0.17046)	NS
*ILSTS019*	7	4.42	0.776	0.681	0.74	0.12382	(0.02715–0.22251)	NS
*ILSTS030*	9	6.01	0.837	0.667	0.81	0.20378	(0.10995–0.29227)	NS
*ILSTS087*	7	3.16	0.686	0.667	0.64	0.02767	(−0.06163–0.11632)	NS
*INRA005*	4	2.58	0.615	0.621	0.55	−0.00962	(−0.12535–0.09767)	NS
*INRA006*	9	7.63	0.872	0.671	0.86	0.23073	(0.13451–0.31479)	NS
*INRA023*	7	5.73	0.828	0.507	0.8	0.38867	(0.28331–0.48339)	***
*INRA063*	5	2.50	0.601	0.462	0.52	0.23224	(0.10057–0.35608)	NS
*INRA172*	7	3.78	0.738	0.655	0.69	0.11342	(0.01843–0.20403)	NS
*MAF065*	10	4.28	0.769	0.664	0.74	0.13723	(0.03830–0.23089)	NS
*MAF209*	2	1.52	0.341	0.269	0.28	0.21245	(0.02267–0.38747)	NS
*McM527*	9	4.20	0.765	0.669	0.73	0.12542	(0.01993–0.21898)	NS
*MM12*	14	7.46	0.869	0.813	0.85	0.06528	(−0.00962–0.13071)	NS
*OarFCB011*	10	4.58	0.784	0.734	0.75	0.064	(−0.02090–0.15133)	NS
*OarFCB020*	6	1.70	0.414	0.372	0.38	0.0999	(−0.04615–0.23000)	NS
*OarFCB048*	11	4.69	0.789	0.746	0.76	0.05456	(−0.02320–0.13311)	NS
*OarFCB304*	12	4.77	0.793	0.738	0.76	0.06993	(−0.01207–0.15543)	NS
*SPS115*	6	1.93	0.488	0.549	0.4	−0.13716	(−0.27617–0.00121)	NS
*SRCRSP05*	7	5.47	0.82	0.759	0.79	0.07493	(−0.01091–0.15903)	NS
*SRCRSP08*	6	5.07	0.772	0.643	0.73	0.16705	(0.05626–0.26468)	NS
*SRCRSP23*	11	3.69	0.805	0.722	0.78	0.10356	(0.01086–0.18899)	NS
*SRCRSP24*	8	4.33	0.732	0.558	0.69	0.23787	(0.11786–0.33931)	**
*TGLA053*	7	3.58	0.723	0.722	0.68	0.00138	(−0.09825–0.08853)	NS
*TGLA122*	7	3.09	0.679	0.587	0.64	0.13537	(0.02766–0.23688)	NS
	8	4.3	0.71	0.63	0.67	0,10986	(0.08773–0.12549)	

NS: Not Significant; ** *p* < 0.01; *** *p* > 0.001.

## Supplementary Materials

The following are available online at https://www.mdpi.com/2076-2615/10/6/1026/s1, Figure S1: Genotyping of 8 microsatellites using Genotyper 3.7NT software, Table S1: Reynolds’ genetic distances (below the diagonal) and the FST (above the diagonal) between pairs of populations.

## Notes

^4^ List of the members of the BioGoat Consortium: T. Adebambo, University of Agriculture Abeokuta, Nigeria; L.A. Alvarez Franco, Universidad Nacional de Colombia, Sede Palmira, Colombia; M. Amills, CRAG, Universitat Autònoma de Barcelona, Spain. J.A. Aranguren, Universidad de Zulia, Maracaibo-Zulia, Venezuela; D.O Bedotti, INTA, EEA Anguil “Ing. Agr. Guillermo Covas”, Argentina; E. Chacón, Universidad Técnica de Cotopaxi. Ecuador; M.A. Domínguez Martínez, Universidad Autónoma Benito Juárez de Oaxaca. México; S. Dunner, Universidad Complutense de Madrid, Spain; A. Elbeltagy, APRI, Ministry of Agriculture, Cairo, Egypt; A. Galarza, Universidad Mayor de San Simón, Cochabamba, Bolivia; N. Gómez Urviola, ONG CEIN-PUNO. Puno. Perú; J. Jordana, Universitat Autònoma de Barcelona. Spain; M.R. Lanari, INTA EEA, Bariloche, Argentina; A. Manunza, CRAG, Universitat Autònoma de Barcelona, Spain; R. Martínez, Universidad Nacional de Asunción, Paraguay; P.M. Parés i Casanova, Universitat de Lleida. Spain; A. Pons Barro, SEMILLA, Islas Baleares, Spain; M.A. Revidatti, Universidad Nacional del Nordeste, Argentina; M.N Ribeiro, Universidade Federal Rural de Pernambuco, PE, Brasil; A. Abu Shady, Ain Shams University. Cairo, Egypt; B. Seguí, Consejería de Medio Ambiente, Gobierno Balear, Spain; P. Sponenberg, Virginia-Maryland Regional College of Veterinary Medicine, Virginia Tech, USA; A. Stemmer, Universidad Mayor de San Simón, Cochabamba, Bolivia; Oriol Vidal, Universitat de Girona, Spain.
