# Supplementary material for: Genetic Characterization of the “Chusca Lojana”, a Creole Goat Reared in Ecuador, and Its Relationship with Other Goat Breeds
Source: Animals (Basel). 2020 Jun 12;10(6):1026. doi: 10.3390/ani10061026 (PMC7341184; doi:10.3390/ani10061026)
Supplement: Supplementary file 1 [file animals-10-01026-s001.zip › SUPPLEMENTARY FILE.pptx]

## Slide 1
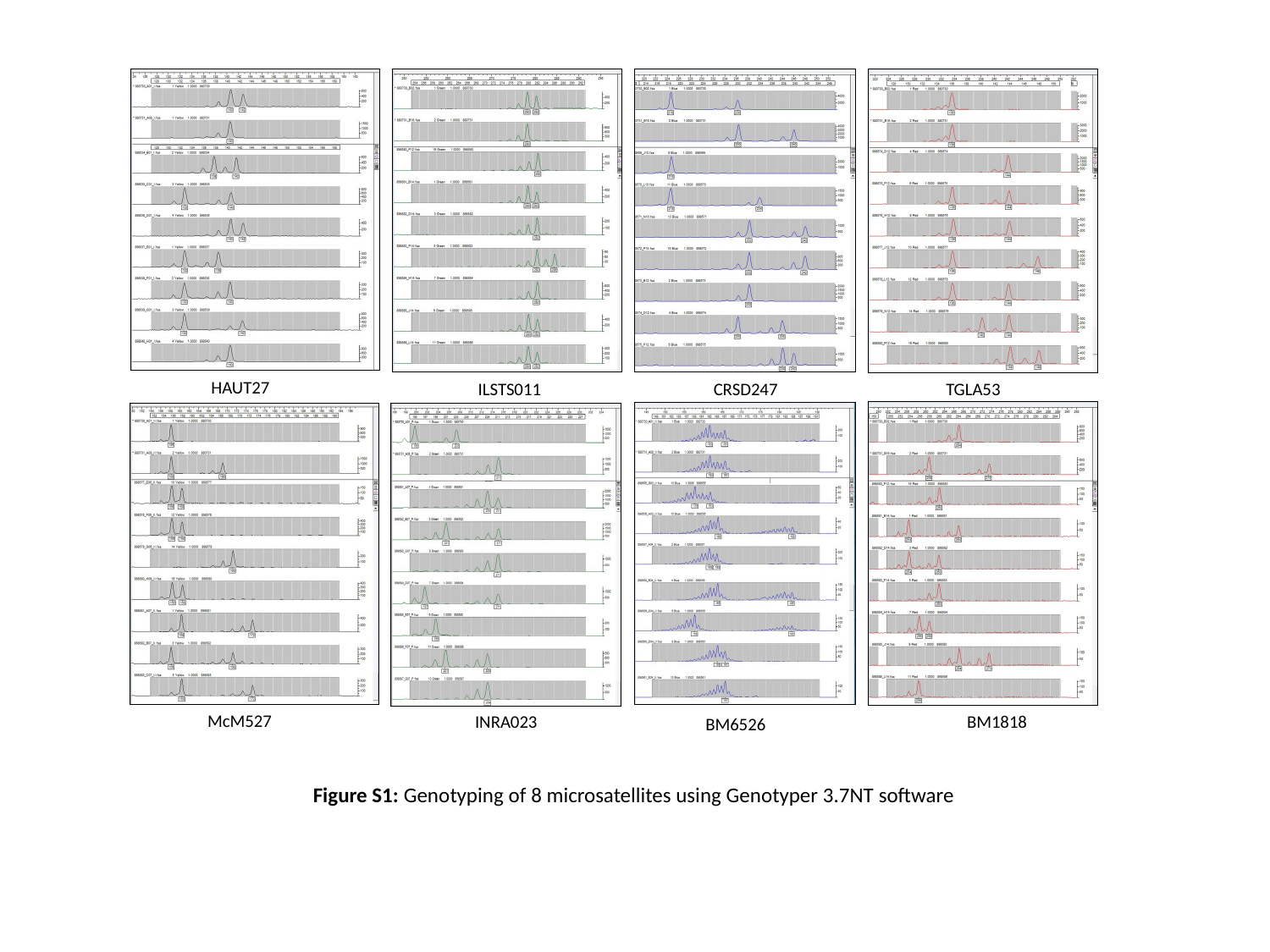

HAUT27
ILSTS011
CRSD247
TGLA53
McM527
INRA023
BM1818
BM6526
Figure S1: Genotyping of 8 microsatellites using Genotyper 3.7NT software

## Slide 2
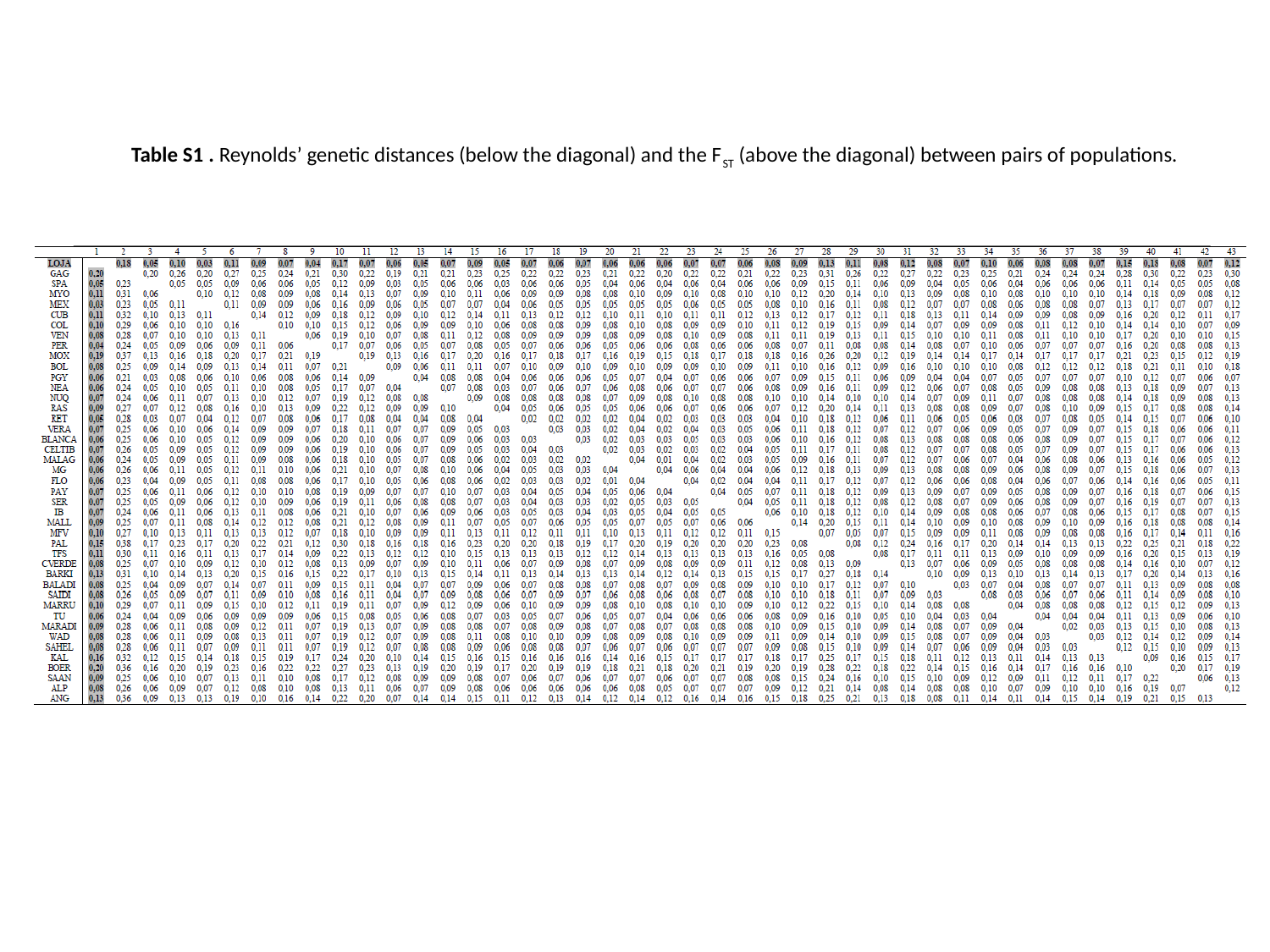

Table S1 . Reynolds’ genetic distances (below the diagonal) and the FST (above the diagonal) between pairs of populations.
